# Transforming healthcare: A pilot study to improve primary healthcare professionals’ self-management support behaviour through blended learning

**DOI:** 10.1186/s12909-024-05799-z

**Published:** 2024-07-30

**Authors:** Lotte Timmermans, Peter Decat, Veerle Foulon, Ann Van Hecke, Mieke Vermandere, Birgitte Schoenmakers, the Primary Care Academy

**Affiliations:** 1 Academic Centre of General Practice, Kapucijnenvoer 7, Kapucijnenvoer 7 - , Box 7001, 3000 Louvain, Louvain, KU Belgium; 2 General Practice and Primary Health Care, Ghent University, Ghent, Belgium; 3 Clinical Pharmacology and Pharmacotherapy, KU Leuven, Louvain, Belgium; 4 University Centre for Nursing and Midwifery, Ghent University, Ghent, Belgium; 5 Department Nursing Director, Ghent University Hospital, Ghent, Belgium

**Keywords:** Self-management, Primary healthcare, Educational intervention, Kirkpatrick evaluation model

## Abstract

**Background:**

Self-management of a chronic condition is a complex but increasingly important issue. However, a supportive attitude and behaviour among healthcare professionals is hampered by a lack of awareness, knowledge and motivation. In addition, the role of professionals in supporting self-management seems unclear.

**Methods:**

A blended learning program for primary healthcare professionals was developed to strengthen self-management support in primary care. The program was piloted in community health centres and multidisciplinary medical practices in Flanders. Using the Kirkpatrick model, the impact on healthcare professionals’ reaction, learning and behaviour regarding self-management support was evaluated.

**Results:**

A total of 60 healthcare professionals registered for the educational program. Post-learning questionnaires and verbal feedback showed a positive response, with professionals highly appreciating the innovative blended learning approach. In terms of learning, participants showed a good understanding of self-management support, although nuances were observed in the application of acquired knowledge to practice scenarios. Finally, preliminary insights into behavioural change were explored, revealing a positive impact of the intervention on participants’ supportive self-management behaviours in healthcare practice.

**Conclusions:**

Our study provides preliminary insights into the outcomes of a blended learning program designed to increase awareness and knowledge of self-management support among professionals. The program needs to be refined for general implementation in primary care.

**Supplementary Information:**

The online version contains supplementary material available at 10.1186/s12909-024-05799-z.

## Background

Worldwide, our healthcare systems are undergoing continuous and fundamental changes in response to various challenges, particularly to the increasing number of people with chronic conditions [1, 2]. One of the key components of this changing healthcare landscape is the emphasis on self-management support as an essential aspect of healthcare delivery [3]. This focus is not only driven by international policy, as evidenced by WHO recommendations [4]. Self-management support is also receiving increasing attention at national level, in Belgium [5].

Self-management of a chronic condition is defined as “the individual’s ability to manage the symptoms, treatment, physical and psychosocial consequences and lifestyle changes inherent in living with a chronic condition” [3]. Engaging patients in self-managing their chronic condition is beneficial for both health and quality of life [6–8]. Healthcare professionals play a crucial role here [9]. Despite the clear benefits of self-management support, its successful integration into healthcare practice remains a challenge [10, 11]. Barriers include limited awareness and knowledge of the role of healthcare professionals, the persistence of traditional consultation models, the lack of clear guidelines and models, and the limited accessibility and applicability of supportive interventions [12–14]. As the role of the primary healthcare professionals is often underestimated, it is essential to empower this group by focussing on their attitudes and behaviour towards self-management support to achieve effective support in practice [15].

Therefore, our research group conducted an in-depth analysis of professionals’ behaviour regarding self-management support in primary care practices. Using Michie’s behaviour change wheel (BCW) [16], we concluded that to achieve supportive behaviour, there should be a focus on ‘education’ and ‘enablement’ of professionals. Indeed, these two components emerged as the key intervention functions from the behavioural analysis. To integrate both education and enablement, we developed an interactive blended learning intervention.

Blended learning, defined as a mixture of traditional face-to-face and asynchronous or synchronous online learning methods [17], has been shown to increase the effectiveness and flexibility of educational programs. Previous research on blended learning and educational approaches in healthcare has demonstrated its potential to improve the delivery of self-management support by healthcare professionals [18, 19]. Also, recent research shows that blended learning methods, particularly applied to healthcare professionals, are more efficient and lead to greater participant engagement and understanding [20–22]. Moreover, there is a growing need for new online e-learning approaches to meet the demand for high levels of interactivity, reflection, practice and application for healthcare professionals learning to provide effective self-management support, particularly in the context of chronic conditions [19]. Our choice was additionally influenced by the advantages of synchronous and asynchronous learning, giving professionals the flexibility to adapt their learning pace to individual needs and time constraints [18, 23].

Based on these literature findings and on our own behavioural analysis, we developed a high-quality, theory-based intervention called MEnToSS (“More Encouragement Towards Self-management Support”). It’s a blended learning intervention targeted at all types of healthcare professionals and created with input from different stakeholders (patients, informal and formal caregivers, representatives of patient organisations, policy makers, etc.). Detailed information on the intervention is provided in the methodology section.

This paper reports on preliminary insights into the outcomes of the MEnToSS intervention regarding the attitudes and behaviours of healthcare professionals to support self-management. The research question addressed in this paper is: *What is the impact of the MEnToSS learning program on healthcare professionals’ attitudes and behaviours to support self-management?*

## Methods

### Study design

The present study is a pilot study because of its preliminary nature, involving small-scale testing of a completely new intervention. Using a mixed-methods design, the study integrates both quantitative and qualitative research methods to provide an in-depth understanding of the outcomes of the MEnToSS learning intervention. Ethical approval was obtained from the Ethical Committee Research of UZ/KU Leuven (S63890). To ensure transparent reporting, this study follows the CRISP (Consensus Reporting Items for Studies in Primary Care) reporting guideline [24], chosen for their relevance to the primary care context of our study (Supplementary file A).

### Participants

The intervention is tailored for primary healthcare professionals, both health and welfare, in multidisciplinary practices or community health centres. These professionals provide care to a vulnerable population of chronic patients with moderate complex care needs, as defined by Iglesias (2018) [25]. These include people with multimorbidity, medication complexity, increased post-hospital care needs, socio-economic challenges, low health literacy, etc. Participation was limited to teams consisting of a minimum of four healthcare professionals, each of whom represented at least two different healthcare disciplines. Information materials, such as leaflets and a video, were used and distributed through our organisation (i.e., the Primary Care Academy—PCA) to recruit participants. In addition, e-mail invitations were sent to various health- and welfare organisations. The study sought to enrol of at least six centres, comprising minimally 24 healthcare professionals.

### MEnToSS intervention

#### Intervention

 The intervention consisted of a blended learning program that combined asynchronous and synchronous learning. The aim was to educate and enable healthcare professionals to more effectively support self-management in primary care practice. This includes providing professionals with the motivation, knowledge and insights needed to address barriers to supporting self-management in practice.

#### Learning objectives

 The intervention was designed to help participants better understand the concept of self-management, the importance of self-management support and define their role as a healthcare professional in it. In addition, they were encouraged to think critically about their own actions in supporting self-management. These objectives contributed to the overall goal of increasing professionals’ knowledge, attitudes and perceived behaviour towards self-management support.

#### Theory

 The learning program was developed by researchers from the PCA consortium according to Horton’s Absorb-Do-Connect (ADC) model [26], chosen for its emphasis on active engagement, practical application, and seamless integration into real-world contexts. The theoretical underpinnings included data from literature analysis [27], interviews [28], focus groups [29] and nominal group brainstorming sessions [30]. These diverse sources of empirical and theoretical material, which closely examined self-management support in primary healthcare, were systematically integrated in Michie’s behaviour change wheel framework [31]. This evidence-based approach consists of eight steps in three phases to develop sustainable interventions, taking into account input from all actors in the healthcare network (i.e.; patients, informal and formal caregivers, representatives of patient organisations, policy makers). This inclusive process ensured that the intervention was not only theoretically robust but also contextually relevant, focusing on the practical aspects of implementation tailored to specific contexts. The methodological rigour of the development process provided the intervention with a solid foundation for behaviour change.

#### Learning materials

 The MEnToSS intervention offered three different learning materials. First, a written course provided an in-depth understanding of the topic of self-management, including its origins and significance and the crucial role that healthcare professionals play. In addition, there were informative short video clips that briefly addressed challenges and misconceptions related to self-management (support). Finally, the learning materials included podcasts with healthcare professionals that gave insights into the topic of self-management support.

#### Learning strategies

**Fig. 1:**
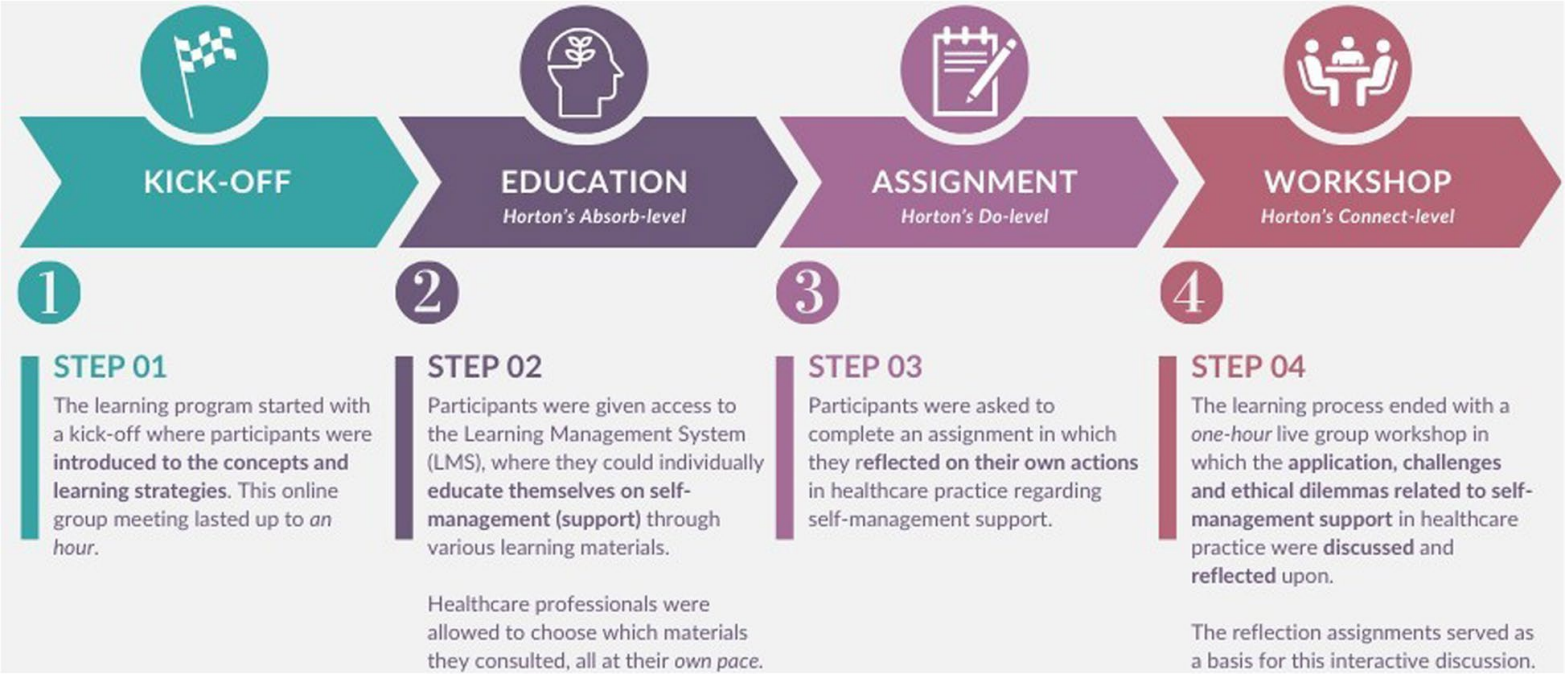
Steps of the blended learning intervention. Template retrieved from ww.canva.com

 The learning intervention consisted of multiple steps, represented in Fig. 1. Participants received a certificate after completion of the learning program.

#### Delivery

The intervention was delivered via a Learning Management System (LMS) (password-protected) over a two-month period, from the starting point to the final workshop. The intervention was initiated at the kick-off and concluded during the final workshop by the main trainer (LT), a primary healthcare researcher and an expert in self-management support. Moreover, this person remained available online throughout the entire learning process to provide ongoing guidance and support. In addition, pedagogical support was received from an assistant trainer with teaching experience (i.e., platform guidance, practical issues, etc.).

It’s important to acknowledge that the main researcher (LT) also facilitated the intervention, acting as both researcher and facilitator/coach. While this dual role offers advantages in terms of research alignment and expert guidance, it also raises awareness of potential biases. Efforts were made to mitigate bias by ensuring that each step of the intervention was rigorously validated and cross-checked by the wider Primary Care Academy team. This collaborative approach helped to minimise bias and ensure the integrity and validity of the intervention.

### Data collection

The evaluation of the MEnToSS self-management support intervention used a mixed-methods approach, incorporating the Kirkpatrick model to assess the levels of reaction, learning and behaviour [32]. This choice was based on previous successful and meaningful applications of the model in different healthcare settings, such as primary care. It allows for a holistic assessment of intervention outcomes, in line with our aim to capture both immediate impressions (learning/reaction) and long-term behavioural change.

Quantitative data were collected using electronic questionnaires (Qualtrics®) to assess program impact or change (Supplementary file B). Responses were evaluated using a 5 or 7-point Likert scale, with the possibility of making additional comments. To evaluate reaction, the Short version of the User Experience Questionnaire (UEQ-S) was distributed immediately after the intervention, complemented by questions on general satisfaction with blended learning (using a modified version of a validated Blended Learning Questionnaire [33]). More specifically, the UEQ-S provides a comprehensive assessment by measuring multiple dimensions of user experience and satisfaction [34]. In addition, the Blended Learning Questionnaire developed by Naaj et al. (2012) assesses learner satisfaction across different aspects of the blended learning experience, including instructor effectiveness, technology integration, and interaction quality [33].

In terms of learning, participants’ self-perceived knowledge and skills related to self-management support were assessed immediately after, with a specific focus on the SILCQ-fundamentals [28]. These fundamentals cover the essential professional tasks of Supporting, Involving, Listening, Coordinating, and asking Questions in primary care practice in order to guide patients towards more effective self-management. These questions, compiled by the research group, included both statements (ranging from strongly disagree to strongly agree) and validations (ranging from very unimportant to very important). Finally, participants’ self-reported behaviour was assessed using another researcher-made questionnaire. These data were collected three months after the intervention. Besides quantitative methods, qualitative verbal feedback was collected. More specifically, participants were invited to share their experiences of the learning trajectory, its impact on their healthcare practice, and any challenges and barriers they encountered in oral feedback sessions. These sessions were organised half an hour before the concluding workshop and were set up as roundtable discussions, using pre-defined questions to encourage in-depth interaction. Discussions were organised by practice/centre, with each group consisting of all participants from the same practice/centre. Although this set-up meant that direct feedback from the concluding workshop could not be included in the oral feedback sessions, this information was collected later through the questionnaires that participants filled in after the learning program. This gave us a full picture of how the intervention was received and allowed us to complete the evaluation.

### Data analysis

Quantitative data from the questionnaires were analysed using SPSS (descriptive statistics). Medians and interquartile ranges were reported when there was noticeable variability and a lack of consistent agreement among respondents, ensuring a comprehensive understanding of professionals’ experiences. Chi-square tests were performed to assess relationships between variables. A *p*-value of 0.05 was considered as the level of significance. For qualitative data from the feedback sessions, thematic analysis was applied, following the approach described by Braun and Clarke (2006) [35]. Input was collected, coded and grouped into themes, which provided insights into participants’ experiences, how the intervention influenced their practice and identified obstacles. We integrated the quantitative and qualitative data using a convergent parallel design, as recommended by Pluye et al. (2018) [36]. This involved analysing the quantitative and qualitative data separately, then comparing and contrasting the findings to identify areas of convergence and divergence, providing a comprehensive understanding of the impact of the intervention.

## Results

A total of 60 healthcare professionals from eight multidisciplinary centres/practices registered for the intervention (Table 1). Of these, 56 participants fully completed the learning program, while four participants (from one centre) dropped out.

**Table 1:** Information on registered multidisciplinary centres/practices

Groups	Type	Number of participants	Background	Note
Group 1	Community health centre	6	1 × psychologist 1 × dietician 2 × GP 2 × nurse	
Group 2	Multidisciplinary GP practice	4	2 × GP in training 2 × GP	Dropped out^a^
Group 3	Community health centre	4	1 × tobaccologist 1 × nurse 2 × GP	
Group 4	Multidisciplinary GP practice	6	1 × nurse 2 × medical secretaries 3 × GP	
Group 5	Community health centre	9	1 × trainee (discipline unknown) 2 × physiotherapists 2 × nurse 4 × GP	
Group 6	Community health centre	8	1 × GP in training 3 × nurse 4 × GP	
Group 7	Multidisciplinary healthcare practice	17	3 × nurse 3 × GP in training 4 × nurse in training 7 × GP	
Group 8	Multidisciplinary GP practice	6	3 × nurse 3 × GP	

^a^Reason: Discontinuation due to engagement challenges (small group size, self-study, podcast length, etc.) and logistical difficulties in scheduling a concluding workshop

### Quantitative user feedback

The first post-intervention feedback questionnaire, focusing on reaction and learning levels according to the Kirkpatrick model, was received from 25 participants. Of these, 72% were female respondents, 28% male respondents, with 64% general practitioners and 24% nurses, as the two largest groups. In terms of learning preference, 24% took an integrated approach to all materials, while 24% combined text with podcasts and 16% took a mix of text and videos. In terms of prior exposure, the initial post-intervention questionnaire revealed that 77% of the participants had previously been exposed to the concept of self-management support, albeit to a very limited extent. This exposure was mainly through formal education and training, focusing on goal-oriented care and positive health. The chi-square test showed no significant relationship between years of experience (*p* = 0.165) or professionals’ background (*p* = 0.829) and previous knowledge of self-management support.

The second feedback questionnaire, which assessed behavioural levels after three months, was completed by 17 participants. Gender and background remained comparable with the first round of feedback, with female respondents and mainly general practitioners or nurses as background.

#### Reaction to the learning materials and learning program

For the written learning materials (referred to as “text”), participants rated their experience on average as 5 out of 7 using the UEQ-S (Table 2).

**Table 2:** Reaction outcomes UEQ-S written materials

Text is	1	2	3	4	5	6	7	Median (IQR)
Obstructive/supportive	0 (0%)	0 (0%)	2 (10%)	3 (15%)	7 (35%)	6 (30%)	2 (10%)	**5 (1.5)**
Complicated/easy	0 (0%)	1 (5%)	2 (10%)	4 (20%)	5 (25%)	7 (35%)	1 (5%)	**5 (2)**
Inefficient/efficient	1 (5%)	1 (5%)	4 (20%)	4 (20%)	6 (30%)	3 (15%)	1 (5%)	**4.5 (2)**
Confusing/clear	0 (0%)	0 (0%)	3 (15%)	4 (20%)	5 (25%)	3 (15%)	5 (25%)	**5 (2.5)**
Boring/exciting	2 (10%)	0 (0%)	4 (20%)	12 (60%)	0 (0%)	1 (5%)	1 (5%)	**4 (1)**
Not interesting/interesting	1 (5%)	1 (5%)	2 (10%)	5 (25%)	7 (35%)	2 (10%)	2 (10%)	**5 (1)**
Conventional/inventive	2 (10%)	1 (5%)	4 (20%)	5 (25%)	7 (35%)	0 (0%)	1 (5%)	**4 (2)**
Usual/leading edge	3 (15%)	1 (5%)	2 (10%)	5 (25%)	7 (35%)	0 (0%)	2 (10%)	**4 (2)**

Left Column: Categories from the Short version of the User Experience Questionnaire (UEQ-S): Obstructive/Supportive, Complicated/Easy, Inefficient/Efficient, Confusing/Clear, Boring/Exciting, Not Interesting/Interesting, Conventional/Inventive, Usual/Leading Edge

Top Row: Participants’ rating (1-7) for written learning materials

Cells: Absolute counts with corresponding percentages in parentheses for each rating

Data is reported as medians and interquartile ranges (IQR)

The interquartile range (IQR) of the dataset was 1. Relatively high scores were given to the level of support (Median (Med) = 5, IQR = 1.5), convenience (Med = 5, IQR = 2), clarity (Med = 5, IQR = 2.5) and interest (Med = 5, IQR = 1). Opinions were divided on the level of efficiency (Med = 4.5, IQR = 2), inventiveness (Med = 4, IQR = 2) and novelty (Med = 4, IQR = 2) and excitement (Med = 4, IQR = 1). Similar results were found for the videos, but the scores were slightly higher. The median score for these visual learning materials was also 5 out of 7 for the UEQ-S (Table 3). The IQR of the dataset was 2.

**Table 3:** Reaction outcomes UEQ-S videos

Videos are	1	2	3	4	5	6	7	Median (IQR)
Obstructive/supportive	0 (0%)	1 (9.1%)	0 (0%)	2 (18.2%)	2 (18.2%)	3 (27.3%)	3 (27.3%)	**6 (3)**
Complicated/easy	0 (0%)	0 (0%)	0 (0%)	1 (9.1%)	2 (18.2%)	5 (45.5%)	3 (27.3%)	**6 (2)**
Inefficient/efficient	2 (18.2%)	0 (0%)	0 (0%)	1 (9.1%)	3 (27.3%)	3 (27.3%)	2 (18.2%)	**5 (2)**
Confusing/clear	0 (0%)	0 (0%)	0 (0%)	2 (18.2%)	1 (9.1%)	4 (36.4%)	4 (36.4%)	**6 (2)**
Boring/exciting	2 (18.2%)	1 (9.1%)	1 (9.1%)	5 (45.5%)	2 (18.2%)	0 (0%)	0 (0%)	**4 (2)**
Not interesting/interesting	2 (18.2%)	0 (0%)	0 (0%)	4 (36.4%)	4 (36.4%)	1 (9.1%)	0 (0%)	**4 (1)**
Conventional/inventive	2 (18.2%)	1 (9.1%)	1 (9.1%)	2 (18.2%)	2 (18.2%)	1 (9.1%)	2 (18.2%)	**4 (4)**
Usual/leading edge	2 (18.2%)	1 (9.1%)	1 (9.1%)	1 (9.1%)	3 (27.3%)	1 (9.1%)	2 (18.2%)	**5 (4)**

Left Column: Categories from the Short version of the User Experience Questionnaire (UEQ-S): Obstructive/Supportive, Complicated/Easy, Inefficient/Efficient, Confusing/Clear, Boring/Exciting, Not Interesting/Interesting, Conventional/Inventive, Usual/Leading Edge

Top Row: Participants’ rating (1–7) for videos

Cells: Absolute counts with corresponding percentages in parentheses for each rating

Data is reported as medians and interquartile ranges (IQR)

High scores were recorded for the level of support (Med = 6, IQR = 3), convenience (Med = 6, IQR = 2 and clarity (Med = 6, IQR = 2). There was a positive trend for level of interest (Med = 4, IQR = 1). Opinions were moderately divided on the level of efficiency (Med = 5, IQR = 2) and strongly divided on inventiveness (Med = 4, IQR = 4) and novelty (Med = 5, IQR = 4).The majority rated the level of excitement as rather neutral (Med = 4, IQR = 2). Finally, the podcasts were rated by the UEQ-S with a median score of 4 out of 7 (Table 4). The IQR of the dataset was 3.

**Table 4:** Reaction outcomes UEQ-S podcasts

Podcasts are	1	2	3	4	5	6	7	Median (IQR)
Obstructive/supportive	2 (13.3%)	0 (0%)	0 (0%)	5 (33.3%)	1 (6.7%)	3 (20%)	4 (26.7%)	**5 (3)**
Complicated/easy	0 (0%)	1 (6.7%)	0 (0%)	2 (13.3%)	5 (33.3%)	3 (20%)	4 (26.7%)	**6 (2)**
Inefficient/efficient	3 (20%)	1 (6.7%)	1 (6.7%)	3 (20%)	4 (26.7%)	2 (13.3%)	1 (6.7%)	**4.5 (2.5)**
Confusing/clear	0 (0%)	1 (6.7%)	0 (0%)	2 (13.3%)	5 (33.3%)	5 (33.3%)	2 (13.3%)	**5 (1)**
Boring/exciting	4 (26.7%)	0 (0%)	3 (20%)	4 (26.7%)	2 (13.3%)	1 (6.7%)	1 (6.7%)	**4 (4)**
Not interesting/interesting	4 (26.7%)	1 (6.7%)	0 (0%)	2 (13.3%)	3 (20%)	4 (26.7%)	1 (6.7%)	**5 (5)**
Conventional/inventive	4 (26.7%)	1 (6.7%)	2 (13.3%)	2 (13.3%)	2 (13.3%)	2 (13.3%)	2 (13.3%)	**4 (5)**
Usual/leading edge	3 (20%)	1 (6.7%)	2 (13.3%)	5 (33.3%)	1 (6.7%)	1 (6.7%)	2 (13.3%)	**4 (3)**

Left Column: Categories from the Short version of the User Experience Questionnaire (UEQ-S): Obstructive/Supportive, Complicated/Easy, Inefficient/Efficient, Confusing/Clear, Boring/Exciting, Not Interesting/Interesting, Conventional/Inventive, Usual/Leading Edge

Top Row: Participants’ rating (1–7) for podcasts

Cells: Absolute counts with corresponding percentages in parentheses for each rating

Data is reported as medians and interquartile ranges (IQR))

High scores were obtained for the level of support (Med = 5, IQR = 3), convenience (Med = 6, IQR = 2) and clarity (Med = 5, IQR = 1). There were different opinions on the level of interest (Med = 5, IQR = 5), inventiveness (Med = 4, IQR = 5) and novelty (Med = 4, IQR = 3). A slightly decreasing trend was observed in the level of efficiency (Med = 4.5, IQR = 2.5) and excitement (Med = 4, IQR = 4).

**Fig. 2:**
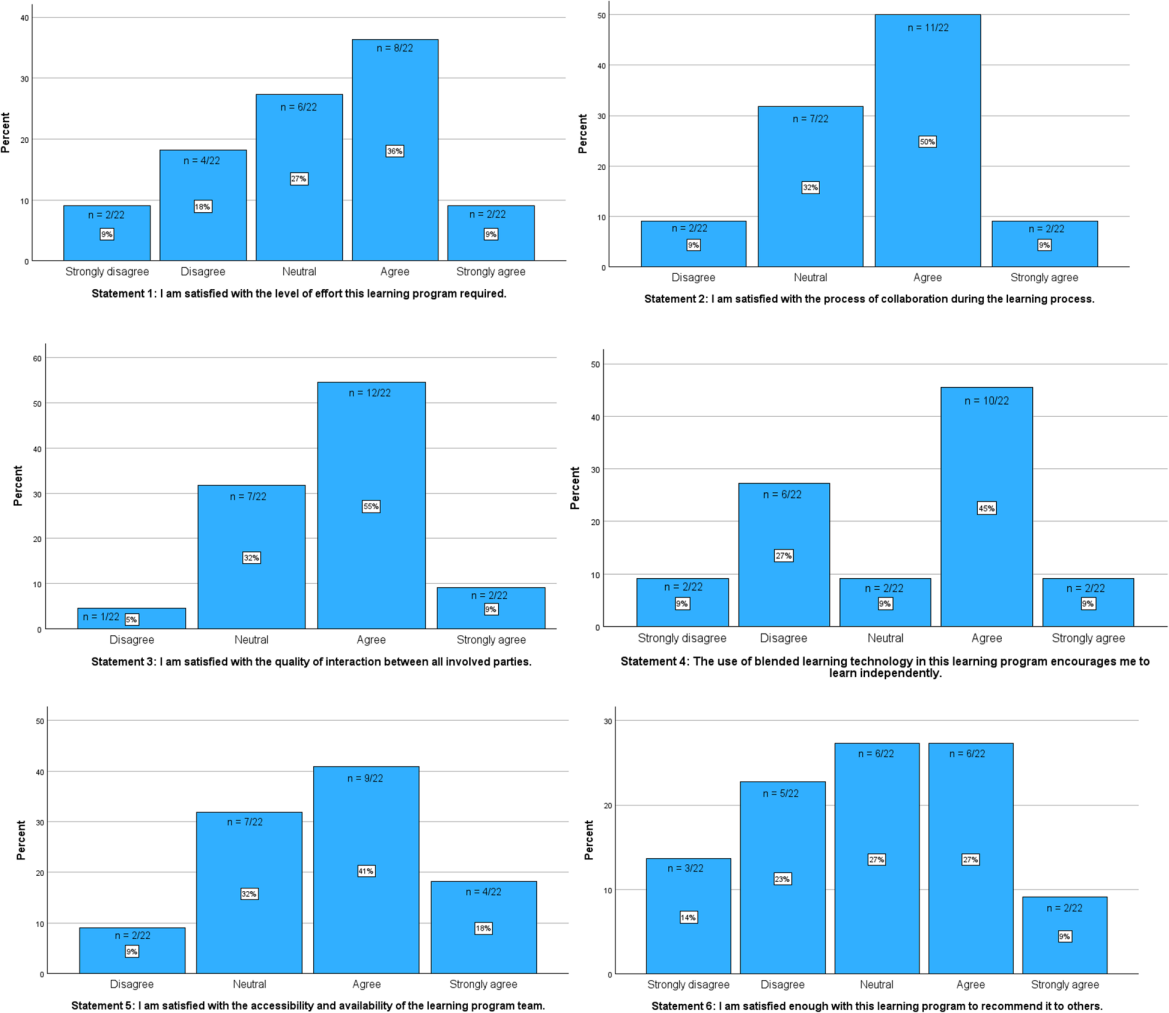
Reaction outcomes post-program questionnaire using statements. X-axis: Agreement Level (Strongly Disagree to Strongly Agree) for statements 1–6. Y-axis: Percentage. Bars represent the distribution of respondents’ agreement levels, with percentages and absolute counts displayed on each bar for each of the six charts

In terms of the learning program in general, the analysis of the modified blended learning questionnaire gave a mixed picture of participants’ experiences (Fig. 2). More specifically, opinions on the perceived effort in getting through the program were strongly divided (statement 1). However, most participants (strongly) agreed that they were satisfied with the level of effort (45%). In contrast, opinions about the collaborative methods during the learning process were positive, with a large majority of participants (strongly) agreeing that they were satisfied with the process of collaboration (59%) (statement 2). In addition, the quality of interaction between all parties involved was also reported as satisfactory by the majority of participants (64%) (statement 3). A mixed picture emerged regarding the use of blended learning technology (statement 4). Although the majority reported to be satisfied (54%), a few participants (strongly) disagreed that the blended technology encouraged them to learn independently (36%). The accessibility and availability of the learning program team was also rated positively by the majority (59%) (statement 5). Finally, opinions were divided on the willingness to recommend this learning program to others (statement 6).

#### Learning about self-management and self-management support

Learning outcomes were examined in the post-program questionnaire using statements and validations grouped into three main categories (Table 5): Relevance of the concepts, including the importance of the SILCQ fundamentals; Knowledge and understanding of the concepts, including being able to unravel misunderstandings; Transferability of knowledge. Medians and interquartile ranges are not presented for the levels of learning and behaviour because participants’ responses to the 5-point Likert scale were remarkably homogeneous. Clear consensus and consistent agreement were observed, resulting in minimal variability between respondents.

**Table 5:** Learning outcomes post-program questionnaire using statements and validations grouped into three main categories: Relevance of the concepts; Knowledge and understanding of the concepts; Transferability of knowledge

		**Strongly disagree**	**Disagree**	**Neutral**	**Agree**	**Strongly agree**
**Relevance of the concepts**	**Statement 7:** I find it important to search with my patients/clients for ways to give disease and health a place in their daily lives	0 (0%)	0 (0%)	0 (0%)	5 (24%)	16 (76%)
	**Statement 8:** I find it important to support selfmanagement of my patients/clients	0 (0%)	0 (0%)	0 (0%)	5 (24%)	16 (76%)
		**Very unimportant**	**Unimportant**	**Neutral**	**Important**	**Very important**
	**Validation 1**: In addition to medical support, space must be made for the provision of practical tools, resources and exchange of information in healthcare practice	0 (0%)	0 (0%)	2 (10%)	10 (50%)	8 (40%)
	**Validation 2:** Care-related choices should be made together with patients/clients in healthcare practice	0 (0%)	0 (0%)	0 (0%)	9 (45%)	11 (55%)
	**Validation 3:** In healthcare practice, space must be made for offering a listening ear	0 (0%)	0 (0%)	0 (0%)	9 (45%)	11 (55%)
	**Validation 4:** Healthcare professionals should take an active role to coordinate care around patients/clients	0 (0%)	0 (0%)	0 (0%)	13 (65%)	7 (35%)
	**Validation 5**: Care professionals should actively ask patients/clients about what is going well, what is not going well, and what their needs are	0 (0%)	0 (0%)	0 (0%)	6 (30%)	14 (70%)
**Knowledge and understanding of the concepts**		**Strongly disagree**	**Disagree**	**Neutral**	**Agree**	**Strongly agree**
	**Statement 9:** I know what self-management is	0 (0%)	0 (0%)	1 (5%)	15 (71%)	5 (24%)
	**Statement 10:** I have insights into self-management	0 (0%)	0 (0%)	1 (5%)	16 (76%)	4 (19%)
	**Statement 11:** I know what self-management support is	0 (0%)	0 (0%)	1 (5%)	16 (76%)	4 (19%)
	**Statement 12:** I have insights into self-management support	0 (0%)	0 (0%)	3 (15%)	13 (65%)	4 (20%)
	**Statement 14:** I understand the importance of selfmanagement support in healthcare practice	0 (0%)	0 (0%)	0 (0%)	11 (52%)	10 (48%)
	**Statement 18**: I have knowledge of the fundamentals of the SILCQ model	0 (0%)	0 (0%)	7 (33%)	10 (48%)	4 (19%)
	**Statement 19:** The learning program has strengthened my knowledge of self-management and selfmanagement support	0 (0%)	3 (14%)	4 (19%)	11 (52%)	3 (14%)
	**Statement 20:** Every person is capable of engaging in self-management to some degree	0 (0%)	1 (5%)	4 (19%)	7 (33%)	9 (43%)
	**Statement 21:** Self-management is achieved in cooperation with your environment	0 (0%)	0 (0%)	0 (0%)	9 (43%)	12 (57%)
	**Statement 22:** Self-management is exclusively about “doing it yourself.”	6 (29%)	10 (48%)	3 (14%)	0 (0%)	2 (10%)
	**Statement 23:** Self-management goes beyond taking responsibility in medical care and treatment	0 (0%)	0 (0%)	0 (0%)	13 (62%)	8 (36%)
	**Statement 24:** Self-management is an innate skill	2 (10%)	13 (62%)	4 (19%)	1 (5%)	1 (5%)
	**Statement 25:** It is the role of healthcare professionals to guide patients/clients to increase self-management	0 (0%)	0 (0%)	2 (10%)	11 (52%)	8 (38%)
**Transferability of knowledge**	**Statement 13:** I can illustrate self-management support with an example from healthcare practice	0 (0%)	0 (0%)	2 (10%)	13 (62%)	6 (29%)
	**Statement 15:** I know in what specific ways I can contribute to self-management support	0 (0%)	1 (5%)	2 (10%)	13 (62%)	5 (24%)
	**Statement 16:** I can explain to my environment what self-management is	0 (0%)	0 (0%)	1 (5%)	16 (76%)	4 (19%)
	**Statement 17:** I can explain to my environment what self-management support is	0 (0%)	0 (0%)	2 (10%)	15 (71%)	4 (19%)

Left Column: Statements 7 – 17 and Validations 1 – 5

Top Row (Statements): Agreement Levels (Strongly Disagree to Strongly Agree)

Top Row (Validations): Importance Levels (Very Unimportant to Very Important)

Cells: Absolute counts with percentages in parentheses representing respondents’ distribution for each statement and validation

##### Relevance

After participating in the learning program, healthcare professionals indicated that they recognised the importance of actively searching for ways to integrate disease and health into patients’ daily lives or the importance of supporting patients’ self-management (statements 7–8: 76% strongly agreed, 24% agreed). The relevance of the fundamentals of self-management support as described in the SILCQ-model was also widely recognised, with an emphasis on involving patients in decision making, listening to them and asking questions (validations 1–5: 30–65% important, 35–70% very important).

##### Knowledge

Participants in the MEnToSS intervention indicated that the learning program contributed significantly to their knowledge (statement 19: 52% agreed, 14% strongly agreed). Also, a significant percentage of participants reported having a good understanding of the concepts, with the vast majority agreeing that they know what self-management is, what self-management support is and what the SILCQ fundamentals are (statements 9, 11, 18: 48–76% agreed, 19–24% strongly agreed). This knowledge extended to insights into self-management and self-management support (statements 10 and 12: 76% agreed, 19% strongly agreed). Misunderstanding and misconceptions about self-management (support) were effectively debunked, with high percentages disagreeing or strongly disagreeing (statements 20, 21, 23 and 25), or agreeing or strongly agreeing in the reversed statements (22 and 24).

##### Transferability

Participants expressed confidence in applying and communicating their knowledge of self-management and self-management support (statements 13, 15–17: 62–76% agreed, 19–24% strongly agreed). They reported a good ability to provide examples from healthcare practice and to articulate specific ways in which they could contribute to self-management support.

#### Behaviour related to self-management support in practice

Three months after the MEnToSS learning program, the majority of respondents in the second questionnaire indicated that they were able to actively search for ways to integrate disease and health into their patients’ daily lives, referring to supporting self-management (statement 26: 82% agreed). In this supportive process, the majority of participants indicated that they were aware of their patients’ social environment (statement 27: 24% were neutral, 75% agreed). Most of them also reported being able to cooperate with this social environment (statement 28: 35% were neutral, 65% agreed). In addition to the informal network, professionals reported having knowledge of the formal health and welfare network around their patients (statement 29: 66% agreed). Collaboration with this network was reported to be more moderate (statement 30: 29% were neutral, 59% agreed).

The five specific self-management support behaviours from the SILCQ-model were also examined. Participants reported being able to provide patients with practical tools, resources and information in addition to medical support (statement 31: 76% agreed, 18% strongly agreed). They also reported being able to make care-related choices together with their patients (statement 32: 76% agreed). Furthermore, respondents indicated they could offer a listening ear in healthcare practice (statement 33: 41% agreed, 53% strongly agreed) and play an active role in coordinating care (statement 34: 76% agreed). Finally, they reported having the skills to actively ask their patients questions about what is going well, what is not going well and what their needs are (statement 35: 65% agreed, 29% strongly agreed). The behavioural results are represented in the appendices (Table 6).

**Table 6:** Behavioural outcomes three months post-program questionnaire using statements

	**Strongly disagree**	**Disagree**	**Neutral**	**Agree**	**Strongly agree**
**Statement 26:** I can search with my patients/clients for ways to give disease and health a place in their daily lives	0 (0%)	0 (0%)	1 (6%)	14 (82%)	2 (12%)
**Statement 27:** I am aware of the social environment of my patients/clients	0 (0%)	0 (0%)	4 (24%)	13 (76%)	0 (0%)
**Statement 28:** I cooperate with the social environment of my patients/clients, subject to their approval	0 (0%)	0 (0%)	6 (35%)	11 (65%)	0 (0%)
**Statement 29:** I know which health and welfare professionals are involved in the care of my patients/clients	0 (0%)	0 (0%)	1 (6%)	15 (86%)	1 (6%)
**Statement 30:** I have contact with the other health and welfare professionals of my patients/clients	0 (0%)	0 (0%)	5 (29%)	10 (59%)	2 (12%)
**Statement 31:** In addition to medical support, I can also offer my patients/clients practical tools, resources and exchange information	0 (0%)	0 (0%)	1 (6%)	13 (76%)	3 (18%)
**Statement 32:** I am able to make care-related choices together with my patients/clients	0 (0%)	0 (0%)	2 (12%)	13 (76%)	2 (12%)
**Statement 33:** I am able to offer a listening ear to my patients/clients	0 (0%)	0 (0%)	1 (6%)	7 (41%)	9 (53%)
**Statement 34:** I am able to take an active role in coordinating care around my patients/clients	0 (0%)	0 (0%)	2 (12%)	13 (76%)	2 (12%)
**Statement 35:** I can actively ask my patients/clients questions about what is going well, what is not going well, and what their needs are	0 (0%)	0 (0%)	1 (6%)	11 (65%)	5 (29%)

Left Column: Statements 26 – 35

Top Row: Agreement Levels (Strongly Disagree to Strongly Agree)

Cells: Absolute counts with percentages in parentheses representing respondents’ distribution for each statement and validation

### Qualitative user feedback

All 56 participants who had completed the MEnToSS intervention provided oral feedback in the structured group discussions at the beginning of the concluding workshop. The intervention received mostly positive responses, with ratings influenced not only by the background of participants, but also by their personality traits, as everyone preferred different learning strategies and methods. The intervention’s emphasis on the person behind the patient was highly appreciated.

“The trajectory provides a fresh perspective, and I have gained more attention [for self-management support].” – Medical secretary

“I have learned to actively listen, observe the whole person, and not just the complaint.” – GP

However, participants with prior knowledge expressed a desire for more profound insights due to overlap with other learning programs. Nevertheless, participants indicated that the MEnToSS learning program added value on several levels. The flexible format of self-study through videos, podcasts and text was seen as a major strength. In particular, the flexibility of self-study allowed participants to progress at their own pace with the learning material that was most appropriate for each individual. As a result, participants indicated that the learning program provided a deeper understanding of the concept of self-management support and its fundamentals (i.e., SILCQ-model). However, the extensive platform also posed challenges, with participants calling for a stronger storyline, more structure and more case-based learning content.

“I open the platform, but if you only have a quarter of an hour, it is not structured enough to motivate you.” – GP

With regard to the learning materials, participants praised the usability and clarity of the podcasts, as well as the short and concise videos, which facilitated independent learning. A minority of participants found the videos too academic and the podcasts boring. To address this, participants suggested making summaries of these learning resources in advance. They wanted to know in advance what would be covered in each video or podcast to assess its relevance, and they also recommended shortening the length of podcast discussions for greater engagement.

“I found the videos concise and, therefore, useful.” – Nurse

The breakdown of self-management support in healthcare practice into five actions, referring to the SILCQ fundamentals, received positive feedback for its simplicity and clear structure. In addition, the assignments on these SILCQ actions, as part of the learning program, were well received. This short, powerful reflection after going through one or more learning materials was perceived as valuable.

“Breaking down the consultation into 5 actions makes it clear and manageable.” – GP

Finally, participants recognised that navigating through the learning program in such an accessible way increased their awareness of the concept and importance of self-management support. The inclusion of take-home messages was described as a possible beneficial added value in the future.

“This intervention opens our eyes to the importance of focusing on these concepts (self-management, self-management support, etc.)” – Nurse

## Discussion

This study aimed to investigate how a blended learning intervention, the MEnToSS intervention, influenced healthcare professionals’ reaction, learning and behaviour towards self-management support in primary care practice. At the reaction level (level 1), participants responded generally positive to the innovative approach of the educational program and recognised the importance of self-management support. The reaction was found to be dependent on several factors, including the learning materials consulted, the participant’s prior knowledge and individual learning style preferences. In general, watching the videos seemed more appealing and, based on the scores, was slightly better received than the other learning materials. Listening to podcasts, in turn, was described as a considerable effort, although those with prior knowledge found them a valuable addition and found more depth in them. This variability highlights the importance of providing varied learning materials and individually tailoring educational interventions to reach and satisfy a wide range of participants [37]. At learning level (level 2), results showed a good understanding of the concept of self-management (support). In addition, the SILCQ-model seemed well understood and was described as a useful tool to reflect on self-management support in practice. At behavioural level (level 3), participants’ confidence to apply the acquired knowledge in their healthcare practice were generally positive. Notably, comparisons between the levels of reaction, learning and behaviour reveals interesting patterns. While the majority had a positive response to the learning program and showed a good understanding of self-management support, responses on knowledge transfer to behaviour in practice showed variation, indicating a possible gap between theoretical knowledge and practical application. Future context analysis should focus on the mechanisms of knowledge transfer to gain deeper insights.

The results of our study are consistent with the conclusion of the systematic review by Collins et al. (2021), which examined the impact of primary care professional education on patient self-management in chronic diseases [18]. Although our study did not directly assess the impact on patients, we also evaluated a professional education program on self-management support. Similar findings emerge, highlighting the critical need for high quality research to investigate the optimal methods and conditions for training primary care professionals. This reinforces our study’s emphasis on nuanced educational approaches. Our learning program differs from previous interventions by using both asynchronous and synchronous learning approaches. We use a variety of learning materials and organise an interactive workshop in a real-life environment. This strategy has been shown to be effective and is in line with the findings of other studies, which show that interactive learning is effective because it reflects the challenges of real-life situations. It promotes deep learning and intrinsic motivation and can potentially bridge the gap between theory and practice [38]. Furthermore, our use of asynchronous components facilitated flexible learning and met participants’ preferences for learning at their own pace and independent of location. The blended strategy benefits from flexibility and self-directed learning and is in line with successful healthcare educational programs that focus on tailored, engaging approaches [37]. The variety of learning materials in our program, including both audio, visual and written materials, improved accessibility and engagement. According to the literature, offering different options accommodate different learning styles, making interventions more inclusive and appealing to a wider audience [39].

Some limitations should be mentioned. First, the use of self-reported outcomes limits the scope of information, preventing a direct translation of findings into practical implications. However, this strategy focuses specifically on the perspectives and experiences of professionals in supporting self-management. Understanding professionals’ perceptions is integral to improving patient self-management support and highlights the importance of their voices in shaping interventions. Secondly, there was no pre-program assessment, which meant that baseline knowledge or behaviour prior to the intervention was not measured. In our case, the decision not to conduct a pre-test was based on the results of an extensive literature review [27], which provided an overview of previous self-management support interventions worldwide. This analysis revealed that our intervention introduced novel approaches and content to self-management support. Consequently, participants’ prior knowledge was likely to be minimal to non-existent, making a pre-test insufficiently justified. In addition, our primary focus was on evaluating the impact of the intervention on real-world outcomes, rather than comparing and measuring improvements in participants’ skills or competencies. In addition, our study encountered a limitation in that we could not use solely standardised questionnaires for evaluation. Given the unique focus on SILCQ, a self-developed model, there were no existing validated customised questionnaires for this purpose. However, we addressed this limitation by using a combination of standardised questions and self-developed measures, with minimal modifications. Finally, it should be noted that the study may be subject to selection bias, as only a small subset of participants who had completed the intervention provided feedback in the questionnaires.

Above limitations highlight the need for cautious interpretation and point to areas for improvement for future evaluations. Therefore, we recommend examining our learning intervention over a longer period of time and with a larger sample size. In addition, future research should explore the nuances of implementation in different primary care contexts to refine and tailor recommendations for maximum impact. Finally, we recommend that future research should go beyond measuring effects on professionals’ self-management support behaviour. It would be valuable to assess the direct effect of our educational intervention in clinical practice by examining how the intervention affects patients’ self-management outcomes. This extension would provide more insight into the wider effects of the intervention and underline its value in practice.

## Conclusions

The MEnToSS intervention not only generated very positive feedback and promoted knowledge acquisition, but also participants perceived the intervention as an opportunity to critically reflect on and more effectively apply the learning to real-life situations in healthcare settings. The success highlights the power of interactive educational programs not only to deliver information, but also to trigger truly transformative learning experiences. As a result, the use of the blended learning approach, with its flexibility and interactive components, suggests that there may be greater awareness of the concept of self-management support among healthcare professionals. This makes the intervention a promising step towards continuous improvement of self-management support in healthcare.

### Supplementary Information

Supplementary material 1.

Supplementary material 2.

## Data Availability

The datasets used and/or analysed during the current study available from the corresponding author on reasonable request.

## Abbreviations

BCW: Behaviour Change Wheel
MEnToSS: More Encouragement Towards Self-management Support
CRISP: Consensus Reporting Items for Studies in Primary Care
ADC: Absorb-Do-Connect
LMS: Learning Management System
UEQ-S: Short version of the User Experience Questionnaire
SILCQ: Supporting, Involving, Listening, Coordinating and Questioning
SPSS: Statistical Package for the Social Sciences
GP: General Practitioner
