# Supplementary material for: Transforming healthcare: A pilot study to improve primary healthcare professionals’ self-management support behaviour through blended learning
Source: BMC Med Educ. 2024 Jul 30;24:823. doi: 10.1186/s12909-024-05799-z (PMC11290224; doi:10.1186/s12909-024-05799-z)
Supplement: Supplementary file 1 — Supplementary material 1. [file 12909_2024_5799_MOESM1_ESM.pdf]

# Appendices

## Supplementary file A

| No. | Reporting Item (Not all items apply to all study designs.) | Included Y | Included N | Included NA | Section | Notes |
|---|---|---|---|---|---|---|
| **1.** | **Include "primary care" and/or discipline-specific terms in the title, abstract, and/or key words.** | ☒ | ☐ | ☐ | I | |
| **2.** | **Describe the study rationale and importance for primary care.** | | | | | |
| 2a. | Explain the rationale for the research question and how it relates to primary care. | ☒ | ☐ | ☐ | I | |
| 2b. | Describe the importance or relevance of the topic under study in the primary care setting. | ☒ | ☐ | ☐ | I | |
| 2c. | Identify any theory, model, or framework used, and explain why it is appropriate to the research question in primary care. | ☒ | ☐ | ☐ | I | |
| **3.** | **Describe the research team's primary care experience and collaboration.** | | | | | |
| 3a. | Describe the research team's expertise and experience in primary care practice and/or research. | ☒ | ☐ | ☐ | M | |
| 3b. | Describe whether and how primary care patients, practicing clinicians, community members, or other stakeholders were involved in the research process. | ☒ | ☐ | ☐ | M | |
| **4.** | **Describe the study participants and populations in the context of primary care.** | | | | | |
| 4a. | Use person-focused language to refer to the research populations and participants, or use terms based on patient preferences. | ☒ | ☐ | ☐ | R | |
| 4b. | If reporting personal characteristics of participants, report the source of the data, the rationale for using it, and the rationale for any classifications used. | ☐ | ☐ | ☒ | R | |
| 4c. | Describe the participants and populations in sufficient detail to allow comparison to other primary care patient populations. | ☒ | ☐ | ☐ | R | |
| 4d. | Specify whether participants have preexisting therapeutic relationships with the clinical team or are new patients. | ☐ | ☐ | ☒ | M, R | **Participants are healthcare professionals.** |
| **5.** | **Describe the conditions under study in the context of primary care.** | | | | | |
| 5a. | Describe whether the condition under study is acute or chronic. | ☒ | ☐ | ☐ | M, R | |
| 5b. | Report how multimorbidity is considered and how it might affect interpretation of the study findings/results. | ☐ | ☐ | ☒ | M | |
| **6.** | **Describe the clinical encounter under study in the context of primary care** | | | | | |
| 6a. | Specify whether the study focus is an isolated clinical encounter or a longitudinal course of care. If it is an isolated clinical encounter, specify whether it is the first visit or a follow-up visit for the condition under study. | ☒ | ☐ | ☐ | M | |
| **7.** | **Describe the patient care team.** | | | | | |
| 7a. | If care is delivered by teams, describe the team members and their roles. | ☒ | ☐ | ☐ | R | |
| 7b. | For each clinician category, report profession, specialty, and qualifications. | ☐ | ☒ | ☐ | R | **Information is not relevant in this small-scale pilot project.** |
| **8.** | **Describe the study interventions in the context of primary care.** | | | | | |
| 8a. | Describe interventions and their implementation in sufficient detail to enable the reader to assess applicability in their own setting. | ☒ | ☐ | ☐ | M | |
| 8b. | Describe any clustering or grouping of patients, participants, clinicians, teams, or practices, and how it was addressed in the analysis. | ☒ | ☐ | ☐ | M, R | |
| 8c. | Describe the health care system in sufficient detail to allow comparisons to other systems. | ☐ | ☒ | ☐ | I, D | **No impact of system on intervention delivery** |
| **9.** | **Describe study measures used and their relevance to primary care.** | | | | | |
| 9a. | Report whether study measurement tools have been validated in primary care populations or settings. | ☒ | ☐ | ☐ | M | |
| 9b. | Describe how the measurement tools used are meaningful to primary care patients and their care. | ☒ | ☐ | ☐ | M | |
| 9c. | Report findings/results to be clinically interpretable by primary care clinicians and patients. | ☒ | ☐ | ☐ | R | |
| **10.** | **Discuss the meaning of study findings/results in the context of primary care.** | | | | | |
| 10a. | Discuss implications of the study findings/results for research, patient care, education, and policy with specific focus on primary care. | ☒ | ☐ | ☐ | D | |
| 10b. | Discuss the implications of study recommendations on demands and priorities in primary care practice. | ☒ | ☐ | ☐ | D | |
| 10c. | Comment on any research processes that might influence the applicability of the study findings/results in diverse primary care settings. | ☒ | ☐ | ☐ | D | |

*Figure 1: CRISP reporting guideline.*

*CRISP = Consensus Reporting Items for Studies in Primary Care; D = discussion; I = introduction; M = methods; N = no; NA = not applicable, R = results; Y = yes.*
