# Supplementary material for: Transforming healthcare: A pilot study to improve primary healthcare professionals’ self-management support behaviour through blended learning
Source: BMC Med Educ. 2024 Jul 30;24:823. doi: 10.1186/s12909-024-05799-z (PMC11290224; doi:10.1186/s12909-024-05799-z)
Supplement: Supplementary file 2 — Supplementary material 2. [file 12909_2024_5799_MOESM2_ESM.pdf]

EVALUATIE VRAGENLIJST

De vragen zijn opgesteld volgens het model van Kirkpatrick. Vragen m.b.t. het niveau tevredenheid van de module educatie (1a-1c) werden geselecteerd uit de gevalideerde User Experience Questionnaire (UEQ). Tevredenheidsvragen over het leertraject in zijn geheel (2) werden geselecteerd uit een gevalideerde enquête ter evaluatie van blended learning programma's. Vragen m.b.t. de niveaus leerresultaat (3) en gedrag (4) kwamen tot stand na doorgedreven literatuuranalyse van onderzoek dat o.b.v. dit model een interventie evalueert en werden inhoudelijk afgestemd op de leerdoelstellingen van het leertraject zelfmanagement ondersteunen. De vragenlijst werd in overleg met onderwijsexperts, gezondheidsprofessionals, kwalitatieve onderzoekers en het eigen onderzoeksteam gefinaliseerd.

**1a. Tevredenheid (verdiepend) cursusmateriaal ("reaction level" Kirkpatrick)**<br>**Na educatieve module 2**								
**Gelieve het online *cursus*materiaal te beoordelen door het aanvinken van een rondje per regel:**	**1**	**2**	**3**	**4**	**5**	**6**	**7**	
Belemmerend	O	O	O	O	O	O	O	Ondersteunend
Complex	O	O	O	O	O	O	O	Eenvoudig
Inefficiënt	O	O	O	O	O	O	O	Efficiënt
Verwarrend	O	O	O	O	O	O	O	Overzichtelijk
Vervelend	O	O	O	O	O	O	O	Spannend
Oninteressant	O	O	O	O	O	O	O	Interessant
Conventioneel	O	O	O	O	O	O	O	Origineel
Gebruikelijk	O	O	O	O	O	O	O	Nieuw

Bron: Schrepp, Martin et al. "Design and Evaluation of a Short Version of the User Experience Questionnaire (UEQ-S)." Int. J. Interact. Multim. Artif. Intell. 4 (2017): 103-108.

**1b. Tevredenheid (verdiepende) kennisclips (“reaction level” Kirkpatrick)**<br>Na educatieve module 2								
**Gelieve de online *kennisclips* te beoordelen door het aanvinken van een rondje per regel:**	**1**	**2**	**3**	**4**	**5**	**6**	**7**	
Belemmerend	O	O	O	O	O	O	O	Ondersteunend
Complex	O	O	O	O	O	O	O	Eenvoudig
Inefficiënt	O	O	O	O	O	O	O	Efficiënt
Verwarrend	O	O	O	O	O	O	O	Overzichtelijk
Vervelend	O	O	O	O	O	O	O	Spannend
Oninteressant	O	O	O	O	O	O	O	Interessant
Conventioneel	O	O	O	O	O	O	O	Origineel
Gebruikelijk	O	O	O	O	O	O	O	Nieuw

Bron: Schrepp, Martin et al. “Design and Evaluation of a Short Version of the User Experience Questionnaire (UEQ-S).” Int. J. Interact. Multim. Artif. Intell. 4 (2017): 103-108.

**1c. Tevredenheid (verdiepende) podcasts (“reaction level” Kirkpatrick)**<br>Na educatieve module 2								
**Gelieve de online *podcasts* te beoordelen door het aanvinken van een rondje per regel:**	**1**	**2**	**3**	**4**	**5**	**6**	**7**	
Belemmerend	O	O	O	O	O	O	O	Ondersteunend
Complex	O	O	O	O	O	O	O	Eenvoudig
Inefficiënt	O	O	O	O	O	O	O	Efficiënt
Verwarrend	O	O	O	O	O	O	O	Overzichtelijk
Vervelend	O	O	O	O	O	O	O	Spannend
Oninteressant	O	O	O	O	O	O	O	Interessant
Conventioneel	O	O	O	O	O	O	O	Origineel
Gebruikelijk	O	O	O	O	O	O	O	Nieuw

Bron: Schrepp, Martin et al. “Design and Evaluation of a Short Version of the User Experience Questionnaire (UEQ-S).” Int. J. Interact. Multim. Artif. Intell. 4 (2017): 103-108.

**2. Globale tevredenheid leertraject (“reaction level” Kirkpatrick)**<br>**Onmiddellijk na einde leertraject**						
	**Sterk mee oneens**	**Oneens**	**Neutraal**	**Eens**	**Sterk mee eens**	**Toelichting**
Ik ben tevreden over de hoeveelheid inspanning die dit leertraject vergde.						
Ik ben tevreden over de manier van samenwerken tijdens het leertraject.						
Ik ben tevreden over de kwaliteit van de interactie tussen alle betrokken partijen.						
Het gebruik van blended learning technologie in dit leertraject moedigt mij aan om zelfstandig te leren.						
Ik ben tevreden over de bereikbaarheid en beschikbaarheid van het leertraject team.						
Ik ben tevreden genoeg over dit leertraject om het aan anderen aan te bevelen.						
	**Vrije input**					
Lijst enkele positieve elementen op na het volgen van het leertraject:						
Lijst enkele verbeterpunten op na het volgen van het leertraject:						

<u>Opmerking:</u>

Bovenstaande vragen komen uit een bestaande gevalideerde Engelstalige vragenlijst m.b.t. evaluatie van blended learning programma’s. Hiervan werd een selectie geïncludeerd op basis van de specifieke beoogde leerdoelstellingen, setting en doelgroep van het zelfmanagement ondersteunend leertraject. Tot slot werden ook twee open vragen toegevoegd.

Bron: Abou naaj, Mahmoud & Nachouki, Mirna & Ankit, Ahmed. (2012). Evaluating Student Satisfaction with Blended Learning in a Gender-Segregated Environment. Journal of Information Technology Education Research. 11. 10.28945/1692.

**3. Leerresultaat leertraject (“learning level” Kirkpatrick)**<br>**Onmiddellijk na einde leertraject**						
	**Ja**		**Nee**		**Toelichting**	
Was u bekend met zelfmanagement voor de start van dit leertraject?						
	**Sterk mee oneens**	**Oneens**	**Neutraal**	**Eens**	**Sterk mee eens**	**Toelichting**
Ik vind het belangrijk om samen met mijn patiënten/cliënten te zoeken naar manieren om ziekte en gezondheid een plaats te geven in hun dagelijkse leven.						
Ik vind het belangrijk om zelfmanagement te ondersteunen van mijn patiënten/cliënten.						
Ik weet wat zelfmanagement is.						
Ik heb inzicht in zelfmanagement.						
Ik weet wat zelfmanagement-ondersteuning is.						
Ik heb inzicht in zelfmanagement-ondersteuning.						
Ik kan met een voorbeeld uit de zorgpraktijk zelfmanagement-ondersteuning illustreren.						
Ik begrijp het belang van de ondersteuning van zelfmanagement in de zorgpraktijk.						
Ik weet op welke manier ik concreet kan bijdragen aan de ondersteuning van zelfmanagement.						
Ik kan mijn omgeving uitleggen wat zelfmanagement is.						
Ik kan mijn omgeving uitleggen wat zelfmanagementondersteuning is.						
Ik heb kennis van de fundamenten van het SILCQ-model.						
Het leertraject heeft mijn kennis over zelfmanagement en de ondersteuning ervan versterkt.						
	**Zeer onbelangrijk**	**Onbelangrijk**	**Neutraal**	**Belangrijk**	**Zeer belangrijk**	**Toelichting**
In de zorgpraktijk moet bovenop medische ondersteuning ook ruimte worden gemaakt voor het aanbieden van praktische tools, hulpmiddelen en uitwisselen van informatie.						
In de zorgpraktijk moeten zorg-gerelateerde keuzes samen met patiënten/cliënten worden gemaakt.						
In de zorgpraktijk moet ruimte worden gemaakt voor het bieden van een luisterend oor.						
Zorgprofessionals moeten een actieve rol spelen om zorg rond patiënten/cliënten te coördineren.						
Zorgprofessionals moeten patiënten/cliënten actief de vraag stellen naar wat goed loopt, wat minder goed loopt, en wat hun noden zijn.						

In hoeverre bent u het eens/oneens met volgende stellingen:						
	**Sterk mee oneens**	**Oneens**	**Neutraal**	**Eens**	**Sterk mee eens**	**Toelichting**
Iedere persoon is in zekere mate in staat om aan zelfmanagement te doen.						
Zelfmanagement doe je in samenwerking met je omgeving.						
Bij zelfmanagement gaat het uitsluitend over "zelf doen".						
Zelfmanagement gaat verder dan verantwoordelijkheid opnemen in medische zorg en behandeling.						
Zelfmanagement is een aangeboren vaardigheid.						
Het is de rol van zorgprofessionals om patiënten/cliënten te begeleiden naar meer zelfmanagement.						

Opmerking:

Bovenstaande stellingen komen direct of indirect aan bod in het leertraject en zijn representatief. Het includeren van misverstanden werd gekozen als strategie omdat het bestaan hiervan mede aan de basis ligt van de ontwikkeling van het leertraject.

**4. Gedrag na doorlopen leertraject (“behaviour level” Kirkpatrick)**<br>**2 maanden na einde traject**						
	**Sterk mee oneens**	**Oneens**	**Neutraal**	**Eens**	**Sterk mee eens**	**Toelichting**
Ik ben in staat om samen met mijn patiënten/cliënten te zoeken naar manieren om ziekte en gezondheid een plaats te geven in hun dagelijkse leven.						
Ik ben op de hoogte van het sociale netwerk van mijn patiënten/cliënten.						
Ik betrek het sociale netwerk van mijn patiënten/cliënten, mits hun goedkeuring.						
Ik weet welke zorg- en welzijnsprofessionals betrokken zijn in de zorg van mijn patiënten/cliënten.						
Ik heb contact met de andere zorg- en welzijnsprofessionals van mijn patiënten/cliënten.						
Ik ben in staat om naast medische, ook praktische ondersteuning en informatie aan te bieden aan mijn patiënten/cliënten.						
Ik ben in staat om zorg gerelateerde keuzes te maken samen met mijn patiënten/cliënten.						
Ik ben in staat om mijn patiënten/cliënten een luisterend oor te bieden.						
Ik ben in staat om een actieve rol op te nemen om zorg rond mijn patiënten/cliënten te coördineren.						
Ik ben in staat om mijn patiënten/cliënten vragen te stellen over wat goed loopt, wat minder goed loopt, en wat hun noden zijn.						
